# Promoting physical activity in a low-income neighborhood of the Paris suburb of Saint-Denis: effects of a community-based intervention to increase physical activity

**DOI:** 10.1186/s12889-016-3360-y

**Published:** 2016-07-29

**Authors:** Camille Buscail, Mehdi Menai, Benoît Salanave, Paul Daval, Marjorie Painsecq, Pierre Lombrail, Serge Hercberg, Chantal Julia

**Affiliations:** 1 Département de Santé Publique, Hôpital Avicenne (AP-HP), F-93017 Bobigny, France; 2 Université Paris 13, Equipe de Recherche en Epidémiologie Nutritionnelle (EREN), Centre d’Epidemiologie et Biostatistiques Sorbonne Paris Cité (CRESS), Inserm U1153, Inra U1125, Cnam, COMUE Sorbonne-Paris-Cité, F-93017 Bobigny, France; 3 Unité de surveillance et d’épidémiologie nutritionnelle (USEN), Institut de veille sanitaire (INVS), Université Paris 13, Centre de recherche en épidémiologie et statistiques, COMUE Sorbonne-Paris-Cité, F-93017 Bobigny, France; 4 Maison de la Santé, 6 rue des Boucheries, F-93200 Saint-Denis, France

**Keywords:** Physical activity, Community-based promotion program, RPAQ, Social inequalities for health

## Abstract

**Background:**

Physical activity (PA) is a key factor for facing the increasing prevalence of obesity and overweight, and should be part of every public health programs. In this context, a community-based public health program promoting PA was developed in a low-income neighborhood of the city of Saint-Denis (France).

**Methods:**

This work aimed at assessing the effectiveness of a 2-year PA promotion program.

A quasi-experimental study was carried out using a pre/post design, with an assessment before (2013) and after (2015) the program. The interviewees were selected using a stratified random cluster sampling. The primary outcome was the proportion of participants practicing sufficient PA (WHO guidelines), and was measured using the RPAQ questionnaire. External interventions (on both neighborhood environment and inhabitants) were listed.

**Results:**

We collected 199 questionnaires at baseline and 217 in 2015. There was a majority of women in both samples: 64.3 % in 2013 and 58.2 % in 2015. The average age of participants was 38.1 years (+/−1.1) and 40.6 (+/−1.1) respectively. The proportion of people practicing sufficient PA was modified from 48.1 % in 2013 to 63.5 % in 2015 (*p* = 0.001). This was mainly driven by women whose level of PA, increased from 40.3 % to 60.3 % (*p* = 0.002), reaching the average national French estimation of PA level among adults (63.5 %).

**Conclusions:**

This work showed a significant increase of the proportion of people practicing PA in a disadvantaged neighborhood where a community-based program promoting PA was developed. Simultaneous external interventions contributed to the results, showing the necessity of synergic interventions to reach efficiency.

## Background

Physical activity (PA) is an important tool for primary or secondary prevention in many chronic conditions. Evidence shows that physically active people, compared to inactive ones, have a lower risk for developing a number of disabling medical conditions and lower rates of chronic diseases like cardiovascular diseases, obesity, colon and breast cancers and even depression [1–8]. Moreover, higher levels of PA have been estimated to reduce by about 30 % the risk for premature all-cause mortality, and a dose–response relationship between PA and health has been highlighted [9]. Despite the positive health effects associated with regular PA, the prevalence of physical inactivity remains high. The World Health Organization (WHO) has estimated that, in 2008, 31 % of adults older than 15 had insufficient levels of PA, and it considers physical inactivity as the fourth risk factor for death worldwide [10]. Furthermore, advantaged populations are more likely to be regularly physically active, less likely to be sedentary and to experience the adverse health outcomes associated with inactive lifestyles than their less advantaged peers [11]. It is indeed thought to be more difficult for low-income groups to access recreational PA facilities such as swimming-pools and sports centers than high-income groups [12–14]. In addition, it is known that the neighborhoods with high walkability encourage active commuting, and the PA level of its residents is therefore quite important [15, 16]. But residents from low-income neighborhoods report less favorable pedestrian/biking facilities, safety from traffic, safety from crime, and therefore less walkability than residents of high-income areas [17].

The city of Saint-Denis in the suburb of Paris (Ile-de-France County, France) is one of the most deprived cities of the country [18] with a high prevalence of obesity and cardiovascular diseases [19–22]. Therefore, it is considered to be a priority area for developing and implementing health promotion programs.

Many studies have investigated the effects of community-based intervention to promote PA [23]. However, only a few of them took place in disadvantaged populations and to our knowledge, no such intervention was conducted in France [3, 24]. The French National Health and Nutrition Program (*Programme National Nutrition Santé)* (PNNS) is a national public health program aiming at improving the health of the general population through nutrition (including diet and PA). Initially set in 2001 for a period of five years, it has regularly been renewed and is now in its third phase. One of the aims of this third phase is to “promote, develop and increase the level of daily PA for all” [25].

In this national context, several programs have been developed and implemented at different levels. Among them, a community-based intervention promoting PA was developed in a low income neighborhood of the city of Saint-Denis, aimed at increasing the level of PA in the population living in this area. This paper focuses on the quantitative evaluation of this community-based intervention promoting PA. The aim of this work was to assess the impact of the intervention on the level of PA of the inhabitants of the study area.

## Methods

### Population and design

The intervention took place in the neighborhood of “Floréal-La Saussaie-La Courtille” (FSC), in Saint-Denis, département of Seine-Saint-Denis (equivalent to a county), France. This neighborhood included 6,622 inhabitants in 2011. People under 15 years of age represented ¼ of the overall population. Overall, 29 % of families were single parent families (compared 24 % in the city of Saint-Denis). Sixty-nine percent of the population in the area were workers or employees. The unemployment rate reached 23 %, (vs. 10 % at the French national level) [21].

The design was quasi-experimental with a pre/post assessment. The assessment at baseline took place from 2^nd^ to 27^th^ of May 2013 and the post-intervention assessment took place from the 18^th^ of May to the 1^st^ of June 2015. The program promoting PA was named “For health, I move in my neighborhood!” (“Pour la santé, je bouge dans mon quartier !”), and started in the summer of 2013, for a 2-year period. The main objective of the intervention was to increase the proportion of adults meeting with the WHO recommendations for PA by at least 20 % [25].

### Primary outcome

The primary outcome was defined as meeting the WHO recommended level of PA for adults, that is : moderate-intensity aerobic (endurance) PA for a minimum of 30 min on five days each week or vigorous-intensity aerobic PA for a minimum of 20 min on three days each week [26, 27]. We used the Recent Physical Activity Questionnaire (RPAQ) validated in French [28] to assess the level of PA of participants. This questionnaire includes items on common means of transportation in everyday life, types and frequencies of several physical activities and type of work (sedentary or physically active). RPAQ describes PA as leisure PA or work PA.

#### Leisure score

We used Metabolic Equivalent of Task concept (MET). The **MET**, or simply metabolic equivalent, is a physiological measure expressing the energy cost of physical activities and is defined as the ratio of metabolic rate (and therefore the rate of energy consumption) during a specific PA to a reference metabolic rate. The more the intense is the PA, the higher the MET is. Values of activities range from 0.9 (sleeping) to 23 (running at 22.5 km/h), moderate PA has a value of 4–5.5 MET and vigorous PA has a MET value of > 6. The total leisure MET for one adult during one week (and therefore his leisure PA level) was measured using the RPAQ. For each leisure activity, the average duration devoted to this activity over a week (estimated from the last four weeks) was multiplied with the corresponding MET and MET-hours for all activities summed to obtain a total leisure MET-hours [29, 30].

$$ Total\  leisure\  score\ \left( Met-\frac{hour}{week}\right)={\displaystyle \sum_{i=1}^n Leisure(i) Met\times leisure(i)\  duration(h)} $$

#### Work score

Depending on the type of work, 4 levels of PA were proposed as follows: sedentary work, low PA work, moderate PA work and intense PA work. In the same way as for leisure physical activity, when someone had a job requiring moderate or intense PA (information collected in the RPAQ), time spent at work was multiplied with the corresponding MET intensity.

#### Global score

Anyone with a total score greater than or equal to 10 MET-hours / week (at least 4 MET, performed 2.5 h per week, corresponding to 30 min of moderate PA, 5 days a week or a job requiring regular moderate or intense PA at least 3 h per week), was considered as meeting the recommended level of PA, and therefore our primary outcome [26, 27].

### Sample size calculation

The French Nutrition and Health Study (*Etude Nationale Nutrition Santé*, ENNS) conducted in France in 2006 using a representative sample has estimated that 60 % of the French population met the moderate recommended level of PA [31]. The objective of a 20 % increase in PA set the intervention as efficient if 72 % of the population reached the primary outcome criteria post-intervention. A priori power calculations were conducted (80 % power in two-tailed tests, alpha 0.05), and determined the sample size for each group at 244. To take into account a cluster effect, this sample size was increased by 20 %, leading to a total of 300 participants. Finally, considering a participation rate at 40 %, 750 participants for each phase had to be approached for participation.

### Sampling method

Individuals were selected for participation in the study using a stratified random cluster sampling among dwellings in the neighborhood. Dwellings of the neighborhood were regrouped in clusters of 4 to 6, spatially close (sharing a stairwell or landing) and clusters were randomly selected. The first adult who met the interviewer and agreed to answer was questioned. Thus, only one adult per household was interviewed.

The neighborhood contained a total of 1,993 apartments which were divided into four strata, as follows: 413 in *La Saussaie*, 440 in *La Courtille*, 650 in *East Floreal* and 490 in *West Floreal*. Every building was divided into clusters. Clusters were distributed as follows: 74 in *Saussaie*, 88 in La *Courtille*, 130 in *East Floreal* and 102 in *West Floreal*.

The number of clusters (and therefore of dwellings) to selected within a stratum was proportional to its size. Clusters investigated were divided as follows: 31 in *Saussaie*, 33 in *La Courtille*, 48 in *East Floréal* and 36 in *West Floréal*. The sampling of clusters were conducted using *alea* function of excel software (Microsoft excel 2010®). Two different samples were selected, one for the baseline assessment (2013) and the other one for the post-intervention (2015).

### Investigation procedure

Questionnaires were administrated to residents of every dwellings contained in the selected clusters using a door-to-door method during the week (from Monday to Friday) between 4 pm and 7 pm. Interviewers rang or knocked at the doors of selected dwellings. In case of two non-responses, they progressed to the next door. The questionnaire was administered face-to-face to the adult who opened the door and accepted to answer. Once all dwellings had been investigated, a second round was conducted in empty clusters (that is the clusters without any questionnaires completed). The survey team included 3 people: 1 public health medical student and 2 students in Master on public health and nutrition. They were trained to standards and requirements for the RPAQ questionnaire completion before the beginning of the field survey.

### Data collection

At baseline and at post-intervention, data were collected on: gender, age, occupation, number of adults in the household, number of children in the household and their age (3–10, 11–14 and 15–17 years). Data on primary outcome (PA) were measured using the RPAQ in French. The questionnaire was completed with information about respondent’s perceptions of the neighborhood in terms of sport facilities, walkability and their sport habits. The study was registered by the French data protection authority (n° CNIL 1665879v0).

### Interventions promoting physical activity

#### Program «For health, I move in my neighborhood!»

From May to August 2013, in parallel to the evaluation of initial level of PA of the population, a qualitative assessment of the barriers and levers to the practice of PA in the neighborhood was conducted among the inhabitants. The results of this study are not developed in this paper which mainly focuses on quantitative assessment, but are detailed elsewhere.^1^ The main barriers identified through this assessment were used to define the main domains (i.e.,: increasing offer of PA, improving communication on PA and changing environment) and therefore the actions to develop in the FSC neighborhood. In August 2013, an instructor in adapted physical activity and health implemented several actions promoting PA through the following three objectives:

##### Improving offering and accessibility to physical activity at community centers

Four new activities were proposed: walking, yoga, fitness and strength training. These activities took place during school time, with affordable costs. Some one-off activities were also proposed (bike outing, orienteering, sports games, etc.).

##### Communication

Flyers and informative brochures were created and Flyers and informative brochures were created and the instructor in adapted PA and health visited different structures of the neighborhood such as schools, community centers or seniors’ residence in order to conduct interventions. These interventions aimed to raise awareness about the benefits of regular PA and balanced diet on health. Over the study period, 43 interventions were conducted, which were followed by 856 people. A permanent service for “reception, information and support for physical activity” was also set in place in several structures of the neighborhood. People who wanted to start, resume or increase their physical activity were able to come and meet the instructor in order to find the most adapted way to meet their objectives. Finally, a “Sports Festival” was created once a year, with sporting events during one day.

##### Environmental changes

Pedestrian orientation paths were developed, with the participation of students and inhabitants of the neighborhood. They aimed to identify the most pleasant and fastest ways to walk to different areas of interest (metro station, center of the town, parks, etc.) and informed residents about the duration and the distance of each itinerary. They were the basis for the implementation of a true pedestrian signage aiming at encouraging people to walk instead of using motorized vehicles for short trips. However to date, pedestrian signage has not been set up in the area.

From the start of the project, there was a strong demand from the inhabitants to benefit from a free access area for practicing sport activities. Thus, six sport devices (such as fitness machines, an elliptic trainer…) were put in place in July 2014. Types of devices and their location in the neighborhood (in the field located in front of the community center) were chosen in agreement with residents. These facilities enable people practicing sport in the area with minimal barriers (accessible at any time and free) and also create a social link between residents through sport.

#### External interventions

##### Urban redevelopment

The FSC neighborhood belongs to priority areas for renovation by the National Agency for Urban Renewal (ANRU). Therefore, several roadworks were conducted during the 2013–2015 period, in particular the redevelopment of the main street leading into the neighborhood, and the redevelopment of a street in the heart of the area. These works included the creation of a bike path, the widening of sidewalks and the installation of speed bumps allowing safer spaces for pedestrians. Other actions can be reported, like the rehabilitation of buildings and the redevelopment of green areas of *Saussaie*. Finally, the neighborhood benefited from the progressive installation of buried containers for the garbage at the foot of the buildings, saving space on the sidewalks, and an improving cleanliness of the area.

##### “Shape and health challenge” program

This program promoting PA was developed by the Sport & Health Association of the city of Saint-Denis and has taken place partly on the FSC neighborhood since 2011. It targets people trying to start PA or start PA again regularly. It lasts 12 weeks, and includes physical and sports activities adjusted to various physical conditions of participants [32].

### Statistics

Sociodemographic features and primary outcome comparisons between the two samples were conducted using Chi-squared tests, weighted Chi-squared tests and logistic regression models. An analysis of variance (ANOVA) was used to compare mean age between the two populations. Given a difference in mean age at borderline of significance between the two samples (*p* = 0.11), analyses on primary outcome were adjusted for age. Interactions between the primary outcome (evolution of PA between 2013 and 2015) and main sociodemographic variables (i.e., age, gender and professional status) were assessed by introducing an interaction term into the models. Logistic regression models were weighted by the inverse of the inclusion probability of each unit (inversely proportional to the number of adults living in the home). All tests of significance were two-sided, and a *p*-value < 0.05 was considered significant. All analyses were conducted using SAS software (9.3 version, SAS institute, Inc., Cary, NC). The PROC SURVEY option was used to take into account the sampling design.

## Results

Overall 741 dwellings were surveyed in 2013, and 738 in 2015. Among the dwellings sampled, 416 questionnaires were collected: 199 were collected at baseline (i.e., a participation of 26.8 %) and 217 were collected 2 years later (i.e., a participation of 29.4 %). Sociodemographic characteristics of the population are described in Table 1. Table 2 presents a comparison of these characteristics with relevant data for the neighborhood derived from of 2011 French census. A non-significant difference in mean age was observed (38.1 years in 2013 vs 40.6 years in 2015, *p* = 0.11). Moreover, compared to the 2011 French census data, women were over-represented (61.5 % vs 52.1 %, *p* = 0.0002) in our samples as well as people aged 45 years and older (38.5 % vs 33.6 % *p* = 0.003).

**Table 1:** Comparison of sociodemographic characteristics between the two samples (*N* = 416) taking into account the sampling design

Variables	2013 assessment (*N* = 199) % or mean (+/−SE)	2015 assessment (*N* = 217) % or mean (+/−SE)	*p* value
Gender			
Male	35.7 %	41.8 %	0.21*
Female	64.3 %	58.2 %	
Age	38.1 (+/−1.1)	40.6 (+/−1.1)	0.11**
Working status			
Employed or student	57.3 %	58.0 %	0.80*
Unemployed	32.3 %	29.8 %	
Retired	10.4 %	12.2 %	
Number of people at home			
1	7.0 %	4.5 %	0.41*
2	17.2 %	15.5 %	
3 or 4	41.5 %	48.0 %	
5 and more	34.3 %	32.0 %	

* Weighted Chi-square tests

** ANOVA model

**Table 2:** Comparison of two samples with the French census data of 2011 (FSC neighborhood)

Population	Together (*N* = 416)	Insee 2011 (*N* = 6622)	*p* value*
Men	38.5 %	47.9 %	0.0002
Women	61.5 %	52.1 %	
% people younger than 45 years	61.5 %	66 %	0.03
% people aged 45 years or more	38.5 %	33.6 %	
Population 15–64 years^a^	377	4150	
Working population employed among 15–64 year-old people^b^	207 (54.9 %)	2258 (54.4 %)	0.85

*FSC* Floréal-Saussaie-Courtille neighborhood

^a^ 2013 and 2015 samples do not include people younger than 18 years. Data represent people aged from 18 to 64 years (and not people aged from 15 to 64 years)

^b^ Anyone employed during the survey time (which excludes housewives, retirees, unemployed persons, students and persons on training)

* Chi-square tests are used to compare sociodemographic features of our study population with French census data of 2011

Results of primary outcomes are presented in Table 3. The proportion of inhabitants reaching a sufficient level of PA was 48.1 % at baseline and 63.5 % at post-intervention. This represents a significant increase of 32.0 % between the two investigation phases (*p* = 0.001). A borderline significant interaction with gender, was observed for leisure score (*p* = 0.06), with a higher increase of the PA level for women. None of the other explored interactions were significant (data not shown). Table 4 presents a comparison of the average walking scores between the pre and the post-assessment. Average walking score from RPAQ was significantly higher in 2015 than in 2013 (*p* < 0.0001), and this increase was higher for women (11.5 vs 5.8, *p* < 0.001) than for men (8.0 vs 4.9, *p* = 0.16). Table 5 presents results for the perception of neighborhood by residents and comparisons between the two phases of the investigation. The proportion of people able to name a sport center or a sport association in the neighborhood increased from 21.9 % in 2013 to 40.6 % in 2015 (*p* = 0.001). Similarly, 54.6 % of interviewees in 2015 vs 30.8 % in 2013 (*p* = 0.12) reported they would practice their sport activity in the neighborhood if possible. On the contrary, only 30.0 % of interviewees who practicing regular sport activity reported practicing it in the neighborhood in 2015, vs 60.6 % in 2013.

**Table 3:** Logistic regressions comparing the proportions of adults reaching the recommended PA level “pre and post intervention”, adjusted for age

At least moderate Physical Activity	2013 (*N* = 199) %	2015 (*N* = 217) %	*p* value*
Global*	48.1 %	63.5 %	**0.001**
Women	40.3 %	60.3 %	**0.002**
Men	62.0 %	67.8 %	0.38
Age < 60 years	46.6 %	66.1 %	**0.001**
Age ≥ 60 years	44.4 %	46.2 %	0.92
Leisure*	42.2 %	57.3 %	**0.001**
Women	35.8 %	56.8 %	**0.001**
Men	53.8 %	58.0 %	0.55
Age < 60 years	41.9 %	59.1 %	**0.001**
Age ≥ 60 years	44.4 %	46.1 %	0.92
Work	9.3 %	8.4 %	0.64
Women	4.6 %	4.6 %	0.72
Men	17.7 %	13.7 %	0.45

* Interaction with gender *p* = 0.06

Numbers in boldface are the p-values whose significance is less than 5%

**Table 4:** Comparison of walking score between 2013 and 2015

	2013 (*N* = 199) mean (+/−SE)	2015 (*N* = 217) mean (+/−SE)	*p* value*
Walking score			
Global**	5.5 (+/−0.7)	10.0 (+/−1.0)	**<0.0001**
Women	5.8 (+/−1.0)	11.5 (+/−1.6)	**<0.001**
Men	4.9 (+/−1.2)	8.0 (+/−1.8)	0.16
Age < 60 years	5.0 (+/−0.9)	9.7 (+/−1.3)	**0.001**
Age ≥ 60 years	8.6 (+/−1.9)	12.3 (+/−3.3)	0.33

* Student test

** Interaction with gender *p* = 0.24

Numbers in boldface are the p-values whose significance is less than 5%

**Table 5:** Chi-square analyses comparing the reported sport access in the neighborhood between pre and post intervention

Questions asked	2013 (*N* = 199) %	2015 (*N* = 217) %	*p* value*
Do you regularly practice sport? (YES)	22.3 %	21.9 %	0.93
If you do, do you practice this activity in the neighborhood? (YES) (*n* = 44/*n* = 41)	60.6 %	30.0 %	**0.01**
If you don’t, do you know if practicing this sport in the neighborhood is possible? (YES)	35.9 %	45.4 %	0.57
If it were possible to practice this sport in the area, would you do it? (YES)	30.8 %	54.8 %	0.12
Do you think that walking around in the neighborhood is easy? (YES)	79.0 %	83.4 %	0.27
Do you think the neighborhood is suitable for practicing sport activity (YES)?	57.8 %	63.3 %	0.23
Can you name a center or an association proposing sport in the area? (YES)	21.9 %	40.6 %	**0.001**
Did you heard about the project named « For health I move in my neighborhood? » (YES)		27.8 %	
If yes, how did you heard about it? (*n* = 59)			
Display		51.2 %	
Web site		0.8 %	
Community center		14.6 %	
Environment/family		12.2 %	
Healthcare professionals		15.4 %	
Medical-sport educator		0.8 %	
Other		13.8 %	

* Weighted Chi-square test

Numbers in boldface are the p-values whose significance is less than 5%

## Discussion

This study shows a significant increase of the proportion of residents practicing moderate PA, in a neighborhood of Saint-Denis where a community-based intervention promoting PA was developed and implemented over a two year period. The proportion observed according to the post-intervention (63.5 %) is quite similar to the average in global French population assessed by the ENNS study in 2006 (63.2 %, national assessment [31]).

This improvement was mainly driven by the increase of PA among women (significant increase of 50 % of the proportion of women practicing sufficient level of PA) and specifically to the leisure component (significant increase of almost 60 %). More precisely, women of our study population appeared to have ‘caught up’ an insufficient PA level between pre and post intervention. Indeed, unlike men whose level of PA in 2013 was close to the national estimation (62 %), that of women was much lower (40.3 %).

The proportion of men reaching the WHO recommendations for PA also increased, but not significantly. Moreover, increase of PA level was not significant for people aged 60 and older.

Many studies have investigated the effects of community-based intervention to promote PA [23]. However, only a few of them took place in disadvantaged populations [3, 24]. Taylor and colleagues carried out a review focusing on populations at risk for inactivity including people with low incomes, members of some ethnic minority groups, and those with disabilities. The overall results concerning low income and ethnic minority groups were limited [3]. More recently, Bock and colleagues showed that among eight studies focusing on persons with low socio economic, four reported positive PA outcomes, but the overall mean net percent change (NPC) was low (NPC = 7.7 % [−6.7 %; 22.0 %]; *p* = 0.248) [24]. When focusing on interventions with positive results on PA, it appeared that a multilevel intervention involving the community at all steps in the design and implementation of the program showed the greatest promise for promoting behavior change [33–37]. Moreover, environmental changes need to be combined with behavioral and social interventions to obtain the desired levels of change in activity rates. Given these scientific results, increased PA we observed after our intervention is probably multifactorial. Below we tried to develop the main explanations for this increase.

### Improving the neighborhood’s « walkability »

Various urban redevelopments realized either by ANRU or by the environmental component of intervention certainly made the neighborhood more walkable, by making it safer and more pleasant. Average walking score from RPAQ was significantly higher in 2015 than in 2013 (Table 4) which is consistent with an improvement in the walkability of the neighborhood [15]. The areas promoting PA are usually a mixture of houses, shops and services (in addition, a supermarket reopened in the neighborhood in May 2013 after 2 years of closure). There is also a good connectivity between streets as well as wide and pleasant sidewalks. The various redevelopments described in this study conform to an environment promoting PA [38].

### Intervention « For health I move in my neighborhood! »

Several points indicate that the intervention has contributed to these results. First of all, the increase of PA is almost exclusively due to leisure activity. Encouraging results in terms of communication about PA offers can also be highlighted. For example in 2015, 40.6 % of the sample was able to mention at least one sport center in the neighborhood, against only 21.9 % in 2013 (*p* = 0.001). Almost a third of the people surveyed (27.8 %) reported having heard about the project. A qualitative evaluation of the intervention was conducted at the end of the program and results were encouraging, particularly those related to the participation of inhabitants to sport activities provided in the neighborhood and use of sport equipments.^1^

### Levers for action identified

Beyond these encouraging results, other means for action were identified and could be used to pursue the improvement of PA in this neighborhood. For example, the proportion of residents declaring they practice regularly a sporting activity did not change between the two assessments (22.3 % in 2013 and 21.9 % in 2015, *p* = 0.93) (Table 5). The activities most frequently mentioned were walking and home sports activities (i.e., fitness, exercise bike, strength training, etc.). Although these activities were taken into account in the measure of PA using the RPAQ questionnaire, they were rarely identified as “real” PA by participants. In addition, the condition of some stairwells, as well as their location inside the buildings, has been clearly identified as obstacles to the use of stairs.^1^ It has been shown that the lighting and proximity of staircases with the entrance of a building are significant determinants of their use by inhabitants [39]. Further, motivational signs to encourage stair use were identified as promising strategies to increase PA [23].

### Strengths

One strength of our study relies on the sampling method which ensures *a priori* the comparability of the two samples. Moreover, when compared to the French census data of the neighborhood, our sample was quite representative of the population of the area. Finally, a power calculation was conducted retrospectively. Given the results on the primary outcome at baseline (48.1 %) and post-intervention (63.5 %), and the number of questionnaires collected (n1 = 199 and n2 = 217), the power of the study was therefore estimated at 86.8 %.

### Limits

Several limitations, resulting from the data collection method must be raised. First, the PA level assessment is based on questionnaires filled out face-to-face, hence we can’t exclude a reporting bias about the level of PA, but we assume it was not differential, given the RPAQ presents a good repeatability and a good reliability for the self-reported level of PA [40]. Nevertheless, a reporting bias could lead to an over-reporting of the level of PA, but in our study, PA level at baseline was much lower than the national average. Furthermore, we can’t exclude the occurrence of a selection bias. Someone who is more interested or involved in PA would be more likely to respond to the questionnaire. In addition, our time slots (16 h00–19 h00) might have induced an under-representation of working people, which potentially practice less PA (due to their professional activity). Nevertheless, regarding employment rate among 15–64 years, our sample is comparable to the French INSEE census data (Table 2). Another limitation may result from the sampling. Indeed, several clusters were surveyed in both assessments (37 % in Floreal Est, 39 % in Floreal Ouest, 42 % in La Courtille and 42 % in La Saussaie), and this wasn’t taken into account for the analyses. However, the sample methodology guarantees a priori the independence of the two samples. Finally, there was no control group, which would have helped taking account for the potential bias introduced in the sampling methodology. But given the type of study (community-based intervention), and the limited geographical area where it took place, we wouldn’t have been able to avoid a “contamination” of the control group by the intervention.

## Conclusion

Low-income populations are more likely than other populations to have chronic diseases related to sedentary lifestyles [11]. Increasing PA levels in these populations holds particular promise for improving health, quality of life and reducing health care costs, and, as a result, a significant public health impact can be achieved. The coordinated actions carried out at different levels in the FSC neighborhood give insights as to effective interventions in these populations.

## Abbreviations

ANRU, National Agency for Urban Renewal; ENNS, National Nutrition & Health Study; FSC, *Floréal-La Saussaie-La Courtille* neighborhood; INPES, National Institute for Prevention Health Education; MET, Metabolic Equivalent of Task; PA, Physical Activity; PNNS, National Nutrition and Health program; RPAQ, Recent Physical Activity Questionnaire; WHO, World Health Organization

## Footnotes

1 « Recherche-action promotion de l’activité physique dans le quartier Floréal-Saussaie-Courtille à Saint-Denis ». Paul Daval et Marjorie Painsecq, Maison de la santé de Saint-Denis. September 2015 [In French].
